# Supplementary material for: Correlation and agreement of regional cerebral oxygen saturation measured from sensor sites at frontal and temporal areas in adult patients undergoing cardiovascular anesthesia
Source: PeerJ. 2022 Sep 15;10:e14058. doi: 10.7717/peerj.14058 (PMC9482766; doi:10.7717/peerj.14058)
Supplement: Supplemental Information 2 [file peerj-10-14058-s002.pdf]

ClinicalTrials.gov PRS DRAFT Receipt (Working Version)
Last Update: 03/21/2022 22:33

ClinicalTrials.gov ID: NCT05087836

Study Identification

Unique Protocol ID: HE641311

Brief Title: Correlation of Cerebral Oxygen Saturation Measured From 2 Sensor Sites: Forehead vs. Temporal

Official Title: Correlation of Regional Cerebral Oxygen Saturation Monitoring by NIRS Between Standard Site vs Alternative Sites of Sensor Position in Adult Cardiac Surgery

Secondary IDs:

Study Status

Record Verification: March 2022

Overall Status: Completed

Study Start: November 1, 2021 [Actual]

Primary Completion: February 28, 2022 [Actual]

Study Completion: March 10, 2022 [Actual]

Sponsor/Collaborators

Sponsor: Khon Kaen University

Responsible Party: Principal Investigator
Investigator: Thepakorn Sathitkarnmanee [tsathitkarnmanee]
Official Title: Associate professor
Affiliation: Khon Kaen University

Collaborators:

Oversight

U.S. FDA-regulated Drug: No

U.S. FDA-regulated Device: No

U.S. FDA IND/IDE: No

Human Subjects Review: Board Status: Not required

Data Monitoring: No

FDA Regulated Intervention: No

Study Description

Brief Summary: Cardiac surgery and neurosurgery may decrease cerebral blood flow leading to cerebral dysfunction. Regional cerebral oxygen saturation (rScO2) monitor via Near-infrared spectometry (NIRS) is recommended for early detection and correction. The standard site of NIRS sensor is forehead area which is impractical in operation with incision at forehead area. The investigators suggest an alternative sensor site at temporal area. The objective of this study is to assess the correlation of rScO2 measured from sensor attached at forehead vs. temporal area.

Detailed Description: Cardiac surgery and neurosurgery may decrease cerebral blood flow leading to neurologic morbidity, e.g., postoperative stroke, delirium, or postoperative cognitive dysfunction. Regional cerebral oxygen saturation (rScO2) monitor via Near-infrared spectometry (NIRS) is thus recommended for early detection and correction. Murkin et al.(2007) conducted a randomized controlled trial in coronary artery bypass graft (CABG) with NIRS monitoring showed that patients who received intervention to avoid rScO2 < 75% of baseline had less major organ morbidity including stroke, and mortality. Senanayake et al.(2012) revealed that NIRS could decrease neurologic complication in patients undergoing ascending aortic replacement with moderate hypothermic circulatory arrest.

The standard site of NIRS sensor is forehead area. There are some types of surgery involving incision at forehead area which makes it not possible to attach sensor at this site. The investigators propose an alternative sensor site at temporal area to be used in such situation.

The objective of this study is to assess the correlation of rScO2 measured from sensor attached at forehead vs. temporal area.

Conditions

Conditions: Cerebral Ischemia

Keywords: NIRS
Regional cerebral oxygen saturation
sensor
forehead
temporal

Study Design

Study Type: Observational

Observational Study Model: Cohort

Time Perspective: Prospective

Biospecimen Retention: None Retained

Biospecimen Description:

Enrollment: 21 [Actual]

Number of Groups/Cohorts: 1

Groups and Interventions

Groups/Cohorts	Interventions
Patient undergoing cardiac surgery	Device: Sensor at forehead area

Groups/Cohorts	Interventions
Each patient will has 2 sets of sensors attached at forehead and temporal area.	NIRS sensor attached at forehead area.<br>Device: Sensor at temporal area<br>NIRS sensor attached at temporal area.

Outcome Measures

Primary Outcome Measure:

1. rScO2
   Regional cerebral oxygen saturation

   [Time Frame: intraoperatively]

Eligibility

Study Population: Patients undergoing elective cardiac surgery

Sampling Method: Non-Probability Sample

Minimum Age: 18 Years

Maximum Age:

Sex: All

Gender Based: No

Accepts Healthy Volunteers: No

Criteria: Inclusion Criteria:

- age =/> 18 y
- undergoing elective cardiac surgery
- American Society of Anesthesiologists (ASA) classification II-III

Exclusion Criteria:

- history of intracranial or carotid vascular disease
- previous surgery at face or brain
- abnormal anatomy of face
- re-do surgery

Contacts/Locations

Central Contact Person: Thepakorn Sathitkarnmanee, MD
Telephone: 66-81-9547622
Email: thepakorns@gmail.com

Central Contact Backup: Sirirat Tribuddharat, MD, PhD
Telephone: 66-81-6205920
Email: sirirat.tribuddharat@gmail.com

Study Officials: Thepakorn Sathitkarnmanee, MD
Study Principal Investigator
Faculty of Medicine, Khon Kaen University, Khon kaen, Thailand

Locations: **Thailand**
Faculty of Medicine, Khon Kaen University
Khon Kaen, Thailand, 40002

Contact: Thepakorn Sathitkarnmanee, MD +66819547622
thepakorns@gmail.com
Principal Investigator: Sirirat Tribuddharat, MD, PhD

IPDSharing

Plan to Share IPD:

References

Citations:

[Study Results] Moerman A, Wouters P. Near-infrared spectroscopy (NIRS) monitoring in contemporary anesthesia and critical care. Acta Anaesthesiol Belg. 2010;61(4):185-94. Review. PubMed 21388077

[Study Results] Murkin JM, Adams SJ, Novick RJ, Quantz M, Bainbridge D, Iglesias I, Cleland A, Schaefer B, Irwin B, Fox S. Monitoring brain oxygen saturation during coronary bypass surgery: a randomized, prospective study. Anesth Analg. 2007 Jan;104(1):51-8. PubMed 17179242

[Study Results] Senanayake E, Komber M, Nassef A, Massey N, Cooper G. Effective cerebral protection using near-infrared spectroscopy monitoring with antegrade cerebral perfusion during aortic surgery. J Card Surg. 2012 Mar;27(2):211-6. doi: 10.1111/j.1540-8191.2012.01420.x. PubMed 22458277

[Study Results] Colak Z, Borojevic M, Bogovic A, Ivancan V, Biocina B, Majeric-Kogler V. Influence of intraoperative cerebral oximetry monitoring on neurocognitive function after coronary artery bypass surgery: a randomized, prospective study. Eur J Cardiothorac Surg. 2015 Mar;47(3):447-54. doi: 10.1093/ejcts/ezu193. Epub 2014 May 7. PubMed 24810757

[Study Results] Zheng F, Sheinberg R, Yee MS, Ono M, Zheng Y, Hogue CW. Cerebral near-infrared spectroscopy monitoring and neurologic outcomes in adult cardiac surgery patients: a systematic review. Anesth Analg. 2013 Mar;116(3):663-76. doi: 10.1213/ANE.0b013e318277a255. Epub 2012 Dec 24. Review. PubMed 23267000

Links:

Available IPD/Information:

U.S. National Library of Medicine | U.S. National Institutes of Health | U.S. Department of Health & Human Services
